# Primary Paratesticular Leiomyosarcoma: A Case Report and Literature Review

**DOI:** 10.1155/2020/8827214

**Published:** 2020-09-03

**Authors:** Zeineb Naimi, Semia Zarraa, Salma Kammoun, Safia Yahyaoui, Maha Driss, Chiraz Nasr

**Affiliations:** ^1^University of Tunis El Manar, Faculty of Medicine of Tunis, 1007 Tunis, Tunisia; ^2^Department of Radiation Oncology, Salah Azaiez Institute, 1006 Tunis, Tunisia; ^3^Department of Pathology, Salah Azaiez Institute, 1006 Tunis, Tunisia

## Abstract

Paratesticular soft tissue sarcomas are very rare malignant mesenchymal tumors. With only few cases reported in the literature, data regarding diagnostic and management of these tumors are limited. We reported a case of primary paratesticular leiomyosarcoma in a 72-year-old man complaining of a progressively growing painless right scrotal mass. The patient underwent radical inguinal right orchiectomy and adjuvant 3D conformal radiotherapy to the tumor bed including the surgical scar. The prescription dose was 54 Gy, and no pelvic irradiation was performed. He remained free of recurrence for the last 16 months.

## 1. Introduction

Soft tissue sarcomas of the genitourinary tract are rare malignant tumors accounting for less than 2% of all urological tumors [1, 2]. More specifically, primary sarcomas of the paratesticular tissue are very uncommon with only around 500 cases described in the literature [3, 4]. Due to their low prevalence, medical research remains difficult, and prospective trials are unfeasible. Literature data only consist of few individual case-reports and very small heterogeneous series. The most commonly reported histological subtypes are liposarcoma, followed by leiomyosarcoma, and rhabdomyosarcoma [4]. Here, we report a case of primary paratesticular leiomyosarcoma in a 72-year-old man who underwent radical right orchiectomy and adjuvant radiotherapy.

## 2. Case Presentation

**Figure 1:**
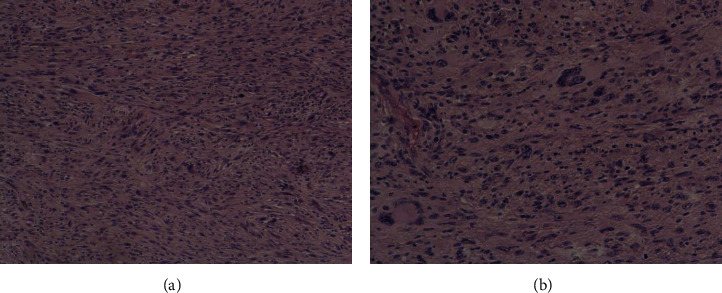
Spindle cell proliferation arranged in interlacing fascicles (×100) (a), with marked cytonuclear atypia at high magnification (×200) (b).

**Figure 2:**
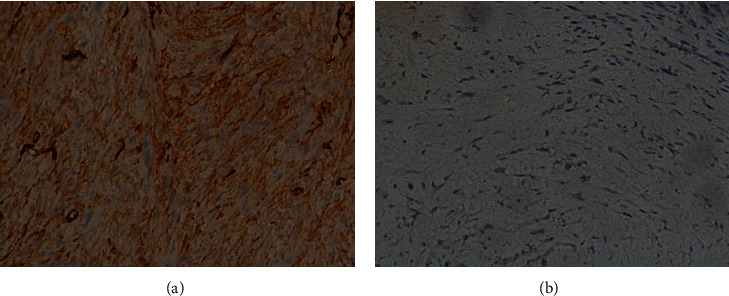
Immunohistochemistry findings showing positive staining for caldesmon (a) and myogenin (b) negativity. Original magnification ×200.

**Figure 3:**
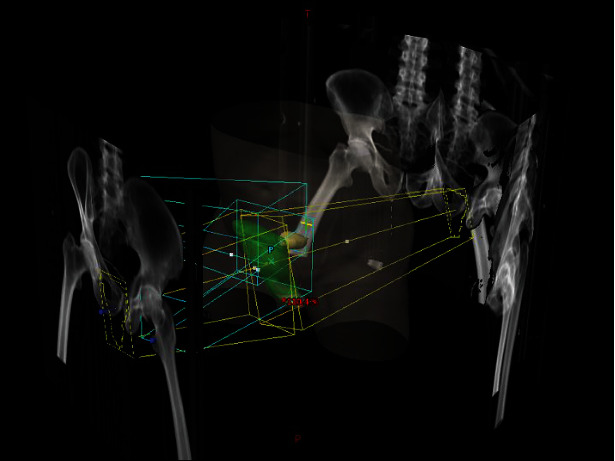
3D reconstruction of radiotherapy beams.

A 72-year-old man with past medical history of hypertension and atrial fibrillation presented to the hospital with a progressively growing painless swelling in his right hemiscrotum as a chief complaint. No lower urinary tract symptoms were reported. Physical examination revealed a 3 cm firm-to-hard mass, isolated from the epididymis. Right epididymis and testis were normal. Scrotal ultrasound revealed a 4 × 3 right extra testicular heterogeneous lesion with peripheral vascularity. The patient underwent radical right inguinal orchiectomy. Grossly, the resected specimen measured 9 cm, showing a 3 cm testis covered by intact tunicae and a distant white surface mass measuring 2 cm. Four satellite nodules were associated, ranging from 2 to 5 mm. Combined histological examination and immunohistochemical study revealed a primary paratesticular grade 1 (score3) leiomyosarcoma arising from the spermatic cord (Figures 1–2). Resection margins were negative. Metastatic workup included a thoracic-abdominal-pelvic computed tomography and showed no distant metastases. The patient was planned for adjuvant 3D conformal radiotherapy to the primary tumor bed (Figure 3). He was clearly informed about the risk of radiation induced infertility and did not wish for sperm banking. Planning CT scan was performed in supine position, with both arms on the chest and legs separated with a diamond-shaped device. CT images were not contrast enhanced and were acquired with a 3 mm slice thickness. Treatment planning was performed using the Varian Eclipse® treatment planning system version 13.7. Clinical target volume (CTV) was defined by the right scrotal and inguinal regions and included the surgical scar. Planning target volume (PTV) was defined by a 10 mm CTV expansion. No pelvic irradiation was performed. The prescription dose was 54 Gy delivered in 2 Gy single daily fraction, 5 fractions per week. Radiotherapy was delivered with 6 and 18 MV photon beams generated by the Clinac iX® Varian linear accelerator. The treatment plan was optimized using beam modifiers such as wedges, angles, collimator angles, multileaf collimators (MLC), and field-in-field technique in order to respect the organ at risk (bladder, rectum, femoral heads, penile bulb, and contralateral testis) dosimetric constraints. Radiotherapy was well tolerated, and no radiation dermatitis was reported. The patient was regularly followed-up for 16 months, with physical examination and scrotal ultrasound every 3 months and annual chest CT. He remained free of local recurrence and distant metastases for the last 16 months.

## 3. Discussion

Paratesticular leiomyosarcoma are unfrequently encountered malignant soft tissue tumors that may arise either from the epididymis, the scrotum, or the spermatic cord [5, 6]. Few cases have been reported in the literature with peak incidence in the 6^th^ and 7^th^ decades and survival rates ranging from 50% to 80% [7, 8]. Nazemi et al. have recently published a large population-based analysis of genitourinary sarcomas [4]. Authors reported a median overall survival of 130 months for paratesticular sarcomas with rhabdomysarcomas showing the best survival. Although little is known about the clinicopathologic features of these neoplasms, tumor grade seems to be the most important prognostic factor and has been related to both local recurrence and distant metastases [9, 10].

Typically, paratesticular leiomyosarcoma presents as a firm nontender scrotal painless mass, independent from the testis on physical examination. Scrotal ultrasound is the primary imaging modality for the evaluation of a scrotal mass, differentiating extratesticular from intratesticular lesions and dismissing several differential diagnoses such as epididymitis, cysts, and hydrocele [11]. However, with no specific imaging features, preoperative diagnosis remains difficult, and histological examination of the resected specimen is required for definitive diagnosis [12].

Due to the rarity of these malignancies, literature data regarding the optimal therapeutic management are inconclusive. However, most of the authors recommend radical orchiectomy with high ligation of the spermatic cord as the standard surgery [13]. Adjunctive prophylactic nodal dissection is rarely performed. With reported nodal involvement rate of up to 29% [14], Banowsky et al. investigated the role of extensive retroperitoneal nodal dissection in a series of 101 patients. No survival benefit was reported [15].

Adjuvant management of paratesticular leiomyosarcoma remains controversial. The Memorial Sloan-Kettering Cancer Center released one of the largest retrospective studies on urologic sarcomas, including 131 patients, of whom 57 presenting with paratesticular localisation [1]. However, data regarding adjuvant treatment were insufficient and therefore not included in analysis. Earlier reports showed high rates of local failure with surgery alone, suggesting the potential benefit of adjuvant radiotherapy in local control improvement [16, 17]. In a series of 18 patients from Massachusetts General Hospital, five of nine patients treated with orchiectomy alone developed locoregional failure, whereas no relapse was reported for the irradiated group [16]. Nonetheless, the median follow-up was shorter for the irradiated group (63 versus 123 months). Similar findings were reported by a retrospective analysis of 21 adult paratesticular sarcomas, showing significantly higher 5-year disease-free survival rate for patients receiving adjuvant radiotherapy (100% versus 56%; *p* < 0.01) [17]. Data regarding the optimal dose are lacking and reported radiotherapy modalities are inconsistent. The prescription dose ranged from 48 to 64 Gy, and pelvic irradiation was not systematically performed [10, 17]. By analogy with the efficacy of radiation in the management of soft tissue sarcomas in general, adjuvant radiotherapy should be considered for paratesticular leiomyosarcoma, especially for patients with high grade tumors and positive margins. Except for pediatric rhabdomyosarcoma, systemic therapy has not shown any strong evidence of benefit in the management of soft tissue sarcoma [18]. Therefore, adjuvant chemotherapy has been rarely considered for paratesticular sarcomas.

## 4. Conclusion

With limited available data, the diagnostic and treatment of paratesticular leiomyosarcoma remains challenging. Although radical inguinal orchiectomy is the mainstay of treatment, adjuvant radiotherapy may improve recurrence-free survival and should be considered, particularly for high-grade tumors. Multi-institutional collaboration and a centralized case management approach are needed to gain experience and better define the optimal management of these tumors.

## Data Availability

All data analysed during this study are included in this published article.
